# Exergoeconomic analysis and optimization of wind power hybrid energy storage system

**DOI:** 10.1038/s41598-024-63247-w

**Published:** 2024-05-31

**Authors:** Caifeng Wen, Yalin Lyu, Qian Du, Boxin Zhang, Xuhui Lian, Qiang Wang, Hongliang Hao

**Affiliations:** 1 College of Energy and Power Engineering, Inner Mongolia University of Technology, Hohhot, China; 2 Key Laboratory of Wind Energy and Solar Energy Technology, Ministry of Education of China, Hohhot, China; 3 Peking University Ordos Research Institute of Energy, Ordos, China

**Keywords:** Wind energy, Batteries, Fluid dynamics

## Abstract

The hybrid energy storage system of wind power involves the deep coupling of heterogeneous energy such as electricity and heat. Exergy as a dual physical quantity that takes into account both 'quantity' and 'quality, plays an important guiding role in the unification of heterogeneous energy. In this paper, the operation characteristics of the system are related to the energy quality, and the operation strategy of the wind power hybrid energy storage system is proposed based on the exergoeconomics. First, the mathematical model of wind power hybrid energy storage system is established based on exergoeconomics. Then, wind power experiments of three forms of thermal-electric hybrid energy storage are carried out, and RSM is used to analyze the power quality and exergoeconomic characteristics of the system, and the optimal working conditions of the experiment are obtained. Finally, an optimization strategy is proposed by combining experiment and simulation. The system efficiency, unit exergy cost and current harmonic distortion rate are multi-objective optimization functions. The three algorithms evaluate the optimal solution based on standard deviation. The results show that the exergoeconomics can effectively judge the production-storage-use characteristics of the new system of ' wind power + energy storage'. The thermal-electric hybrid energy storage system can absorb the internal exergy loss of the battery, increase the exergy efficiency by 10%, reduce the unit exergy cost by 0.03 yuan/KJ, and reduce the current harmonic distortion rate by 8%. It provides guidance for improving the power quality of wind power system, improving the exergy efficiency of thermal-electric hybrid energy storage wind power system and reducing the unit cost.

## Introduction

Human society has gradually deepened the concept of sustainable development, as well as the importance of energy security and ecological environment. This has led to widespread recognition around the world of the need to reduce fossil fuel consumption and promote the development and use of renewable energy^1^. In this context, wind power, as a mature and environmentally friendly renewable energy, recently has been widely developed and applied in the world^2^, and has achieved remarkable results^3–5^. However, wind power is greatly affected by the resource environment, and there are problems such as unstable output^6^ and strong fluctuations^7^, which lead to wind power system overcapacity^8^, insufficient energy^9^ and poor power quality^10^ and other issues. Energy storage can greatly improve the power quality and reliability of the system^11–13^. Therefore, the ' wind power + energy storage ' system can improve the reliability and stability of wind power system.

At present, for the coordinated operation of ' wind power + energy storage ', domestic and foreign experts have carried out a series of exploratory work^14–16^. Li^17^ proposed a wind power-sharing energy storage collaborative primary frequency regulation and capacity optimization strategy considering wind power cluster effect, and analyzed the spatial and temporal correlation of wind speed between wind farms and the impact of wind power cluster effect on primary frequency regulation capacity planning. Moutis P^18^ proposed to adjust the droop control parameters of each fan according to the wind speed, and optimize the power output to improve the primary frequency modulation contribution of the fan. Sun^19^ proposed the frequency regulation control strategy of wind-storage system, and studied the feasibility and economic benefits of large-scale energy storage devices participating in the operation of wind power system. The above literatures apply algorithms such as particle swarm optimization (PSO)^20^, genetic algorithm (GA)^21^, and evolutionary predator and prey strategy (EPPS)^22^ to find the optimal solution of ' wind power + energy storage ' coordinated operation. Although such methods have been widely used in various fields, authors do not consider the unified representation of energy quality in wind power hybrid energy storage systems.

Wind power hybrid energy storage system integrates different energy forms such as heat and electricity. In order to reasonably measure the energy quality, domestic and foreign scholars evaluate the multi-energy coupling system from the perspective of ' quantity ' and ' quality ' based on the exergy analysis method of the second law of thermodynamics. The Dutch Universiteit Twente^23^ systematically introduced different types of exergy in 1997, gave the calculation method of exergy, compared the energy analysis method, and discussed the necessity of exergy analysis method in the field of sustainable development. Compared with enthalpy^24^ analysis method, entropy^25^ analysis method and entransy^26^ analysis method, exergy analysis method^27^ from the perspective of energy quantity and quality^28^ analyzes the conversion, utilization and loss of energy in the process or equipment, which is easier to study the energy quality of different energy forms. Zhang^29^ basing on the exergy analysis method established a unified quantitative characterization method of electricity and heat and a consistent expression of electricity and heat energy consumption, which reasonably reflected the energy quantity of the two products and revealed the way for CHP to exert its energy-saving potential. Meng^30^ proposed a new type of wind-hydrogen coupling system and analyzed system exergy efficiency for the influence of multi-parameters. Caliskan H^31^ applied exergy analysis to improve the evaporator and domestic water heater components of a novel geothermal powered combined cooling, heating and power (CCHP) system. Li^32^ proposed the RIES exergy flow calculation model, and analyzed the RIES exergy flow distribution mechanism for different forms of energy in the regional integrated energy system (RIES). Chen^33^ basing on exergoeconomics established a unified modeling of distributed energy supply system, designed the analysis and evaluation method and process of distributed energy supply system, and took the distributed energy supply system of a power supply bureau as an example for analysis and verification. Han^34^ proposed a new type of full-spectrum solar-assisted methanol cooling, heating and power cogeneration system. Based on the multi-objective optimization model of exergy cost allocation, the comprehensive efficiency of the system was improved. Bushehri M C^35^ basing on a CAES proposed an innovative green hybrid multi-generation system. A comprehensive analysis of the proposed system includes energy, exergy, economic, exergoeconomic, and advanced exergy analyses. Ahamad T^36^ basing on the exergy analysis carried out the multi-objective (Exergy, Energy, Economic and Ecological) optimization of the new solar cogeneration system. Based on the exergy analysis method, the above literatures have made an in-depth study on the comprehensive measurement of energy quality of thermal and electrical hybrid systems, but has not considered the impact of system operating conditions on economy during coordinated operation.

In summary, most scholars at home and abroad^37–41^ use the exergy analysis method to solve the problems of geothermal, solar energy, heat pump and so on. Few scholars put the wind power hybrid energy storage system in the physical environment and the economic environment at the same time to study the energy efficiency and economy of the system. Wen et al. from Inner Mongolia University of Technology carried out a series of studies on the multi-physical field coupling and control technology of permanent magnet wind turbines, the power quality analysis and evaluation of wind turbines, the thermodynamic characteristics and capacity efficiency of wind power systems such as entropy and exergy, and the key technologies of composite energy storage in wind power systems. First, the mathematical model of wind power system exergy analysis is established, and the influence weight of exergy efficiency is determined by analyzing slot type, air gap length, yaw angle, the tip speed ratio, and matching characteristic factors^42^. Considering the influence of the external circuit characteristics on the internal physical field of the motor, the electromagnetic field and temperature field of the generator are precisely solved by the field-circuit coupling method, and the loss distribution of the motor is obtained. By optimizing the air gap length, permanent magnet thickness, and winding conductivity, the best configuration and material properties can improve the efficiency of the motor by up to 4%^43^. Secondly, the system balance and cost conservation model are established, and the efficiency and cost difference are used as the system evaluation index. The sample data of 16 sets of operating conditions are obtained through the central composite design experiment (CCD). Taking the maximum efficiency and the minimum cost difference as the optimization dual objectives, the model is solved by the combination of RSM and NSGA-II, and the optimal operating conditions are obtained^44^. Thirdly, a source-storage-load experimental platform is built, and the test results are analyzed in the frequency domain by combining the wavelet transform and power spectrum analysis method to determine the frequency band of the battery^45^.

In order to explore the influence of thermal-electric hybrid energy storage on the output power quality and exergoeconomic characteristics of the system. On the basis of the research group, this study based on exergoeconomics links the operating characteristics of the system with the characterization of energy quality The mathematical model of wind turbine, generator, battery and heat storage tank is established, and the experimental platform of wind power heat-electric hybrid energy storage is built. At the wind speed of 6 m/s-12 m/s and the speed of 100–600 RPM, the exergy efficiency of wind turbine and generator system, the exergy efficiency of battery system, the exergy efficiency of heat storage system and the exergy efficiency of system are obtained. Based on RSM, the exergoeconomic characteristics of the system are analyzed, and the optimization strategy is obtained. The optimal solution of MOGWO algorithm is obtained by taking the system exergy efficiency, unit exergy cost and current harmonic distortion rate as the objective optimization function, and the standard deviation as the measurement basis. Substitute into the simulation system to verify the effectiveness of the strategy. It provides a favorable reference for measuring the energy quality of the wind storage hybrid system, reducing the system exergy loss and exergetic cost, improving the efficiency of the subsystem, improving the energy efficiency of the wind turbine and reducing the exergetic cost.

## System exergoeconomic scheduling model

**Figure 1:**
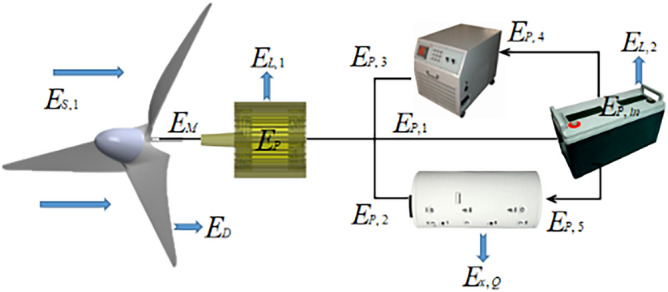
Flow distribution of wind energy storage system.

The wind-storage hybrid system is a complex system that converts heterogeneous energy such as wind energy, mechanical energy, magnetic energy, and electric energy to solve the problem of energy conversion between different forms. In this paper, the concept of exergy is introduced. As an energy measurement unit, exergy according to the available energy is divided into different energy levels, and the heterogeneous energy in the system is uniformly represented. The flow exergy flow distribution of wind power hybrid system is shown in Fig. 1.

Among them,$E_M$ is the wind energy exergy carried by incoming wind,$E_D$ is the exergy flow lost by the wind wheel,$E_M$ is the mechanical energy exergy output by the wind wheel;$E_{P,1},\,E_{P,2},\,E_{P,3}$ is the electric energy exergy of the generator output battery, heat storage system and load,$E_{L,1}$ is the internal exergy loss of the motor;$E_{P,5},\,\,E_{P,6}$ is the electric energy exergy of battery output load and heat storage system;$E_{L,2}$ is the internal loss of the battery,$E_{P,in}$ is the electric energy exergy inside the battery.$E_{x,Q}$ is the exergy loss of heat storage system.

### Objective function

In this paper, the exergy efficiency, economic characteristic index (unit exergy cost) and power quality index (current harmonic distortion rate) of wind-storage hybrid system are analyzed and optimized to improve the exergy efficiency of the whole system.

#### System exergy efficiency

The wind-storage hybrid system is a non-equilibrium dissipative system, and there is an irreversible exergy loss in the process of heat-work conversion. In order to improve the exergy efficiency of the system, the output exergy of each subsystem is the input exergy of the next subsystem, and the exergy loss of the subsystem is reduced^46^. This paper defines the exergy efficiency and exergy loss of each subsystem:

Exergy efficiency of wind turbine system is by Eq. (1):

1 $${\eta _{1}} = \frac{{E_{M} }}{{E_{{S,1}} }}.$$

#### Exergy efficiency of generator system

The generator converts the mechanical energy exergy $E_M$ of the wind wheel output into the electrical energy exergy $E_P$ of the output and the exergy balance equation of the generator system is by Eq. (2):

2 $$E_M = E_{L,1} + E_P.$$

Among them,$E_P$ determined by subsequent subsystems is the output electric energy exergy, $E_{L,1}$ is the internal exergy loss of the motor includes stator copper loss, iron loss, excitation loss, electrical additional loss, mechanical loss, etc.

3 $$E_P = E_{P,1} + E_{P,2} + E_{P,3}.$$

exergy efficiency of generator is by Eq. (4):

4 $$\eta_2 = \frac{E_{P,1} + E_{P,2} + E_{P,3}}{{EM}},$$

#### Exergy efficiency of battery system

When the wind speed is large, the system stores the excess power required to exceed the load, so as to improve the energy utilization rate of the system. When the wind speed is small enough to supply load demand, the battery releases electricity to ensure the safe and stable operation of the system. When the battery is charged by Eq. (5):

5 $$E_{P,1} = E_{P,in} + E_{L,2},$$

6 $$E_{L,2} = \int_{0}^{t} {I_{0}^{2} R_{0} dt} .$$

Among them,$I_{0}$ is the battery charge and discharge current;$R_{0}$ is the internal resistance of storage battery;$EL,2$ is the Internal battery loss;$EP,in$ is the electric energy exergy inside the battery; At this time, the battery exergy efficiency is by Eq. (7):

7 $$\eta_3,1 = \frac{E_{P,in}}{{E_{P,1}}} = \frac{E_{P,1} - E_{L,2}}{{E_{P,1}}}.$$

When the battery is discharged by Eq. (8);

8 $$E_{P,in} = E_{P,out} + E_{L,2} = E_{P,5} + E_{P,6} + E_{L,2}.$$

Among them,$E_{P,out}$ is the battery output electric energy exergy. At this time, the battery exergy efficiency is by Eq. (9):

9 $$\eta_3,2 = \frac{E_{P,5} + E_{P,6}}{{E_{P,in}}}$$

#### Exergy efficiency of heat storage system

At ambient temperature T_0_, when the heat provided by the system (T > T_0_) can be converted into heat exergy $E_{x,Q}$ by Eq. (10):

10 $$\delta E_{x,Q} = \left( {1 - \frac{T_0}{T}} \right)\delta Q$$

Among them, T_0_ is the ambient temperature,${\text{K}}$;T is heating temperature of heat storage system, K;$Q$ is the heat absorbed by the heating process of the heat storage system, J. The heat storage system is heated from T_A_ to T_B_, The process exergy loss is by Eq. (11):

11 $$\Delta E_{x,Q} = T_0\left( {\frac{1}{T_B} - \frac{1}{T_A}} \right)Q$$

The exergy efficiency of the heat storage system is by Eq. (12):

12 $$\eta_4 = \frac{E_{x,Q}}{{E_{P,2}}}$$

The system exergy efficiency is by Eq. (13):

13 $$\eta = \prod\limits_{i = 1}^{n} \eta_i = \prod\limits_{i = 1}^{n} {\left( {1 - \frac{E_{D,j}}{{E_{S,j}}}} \right)}$$

Among them, n is the number of system units; $E_{S,j}$,$E_{P,j}$,$E_{D,j}$ are the input exergy, output exergy and exergy loss of the ith subsystem .

#### Unit exergy cost

In this paper, the unit cost $C{\text{i}}$ is selected as the economic characteristic index by Eq. (14):

14 $$C_{{i}} = \frac{T_i + Z_i}{{E_i}}$$

Among them, $T_i + Z_i$ is the total investment cost of the system;$E_i$ is the total exergy value of the system.

#### Current harmonic distortion

In this paper, the key parameter of power quality index^47^, current harmonic distortion rate (THD_i_), is selected as the index of system operation characteristics by Eq. (15).

15 $$THD_{i} = \frac{{_{{\sqrt {\sum\limits_{h = 2}^{\infty } {\left( {I_{h} } \right)^{2} } } }} }}{{I_{1} }} \times 100\%$$

Among them,$I_{h}$ is the hth harmonic current root mean square value.$I_{1}$ is the root mean square value of fundamental current.

#### Constraint condition

##### System equilibrium constraint

In the wind-storage hybrid system, there is an interrelationship between the subsystems. The output of the previous subsystem determines the input of the next subsystem. For example, the wind exergy of the previous subsystem is converted into mechanical exergy, and the generator of the next subsystem converts the mechanical exergy into electrical exergy, that is, the equilibrium constraint equation of each subsystem is obtained by Eq. (16):

16 $$\sum\limits_{i = 1}^{n} {E_{S,i} } = \,\,\sum\limits_{i = 1}^{n} {E_{P,i} + E_{D,i} } ,$$

Among them,$n$-Number of system units;$E_{{{\text{S}},i}}$,$E_{{{\text{P}},i}}$ and $E_{{{\text{D}},i}}$-Input exergy, output exergy and exergy loss of the ith subsystem.

##### Energy storage battery constraints

Because the battery can only carry out the charge/discharge conversion of electric energy, it cannot generate electricity spontaneously. In order to meet this characteristic^44^ is by equation:

17 $$E_{{\text{i}}dc,T} = E_{{\text{i}}dc,0}$$

Among them, $E_{{\text{i}}dc,T}$ and $E_{{\text{i}}dc,0}$-Termination capacity and initial capacity of the battery.

The specific constraints are as follows:

18 $$\begin{gathered} 0 \le P_{ch}(t) \le P_{ch - \max} \hfill \\ 0 \le P_{disch}(t) \le P_{disch - \max} \hfill \\ S_{oc\min} \le S_{oc}(t) \le S_{oc\max} \hfill \\ P_{ch}(t) \cdot P_{disch}(t) = 0 \hfill \\ \end{gathered}$$

Among them,$P_{ch - \max}$ and $P_{disch - \max}$-The maximum charge and discharge power of battery;$Soc\min$ and $S_{oc\max}$-The maximum and minimum charge state values.

## Experimental scheme

**Figure 2:**
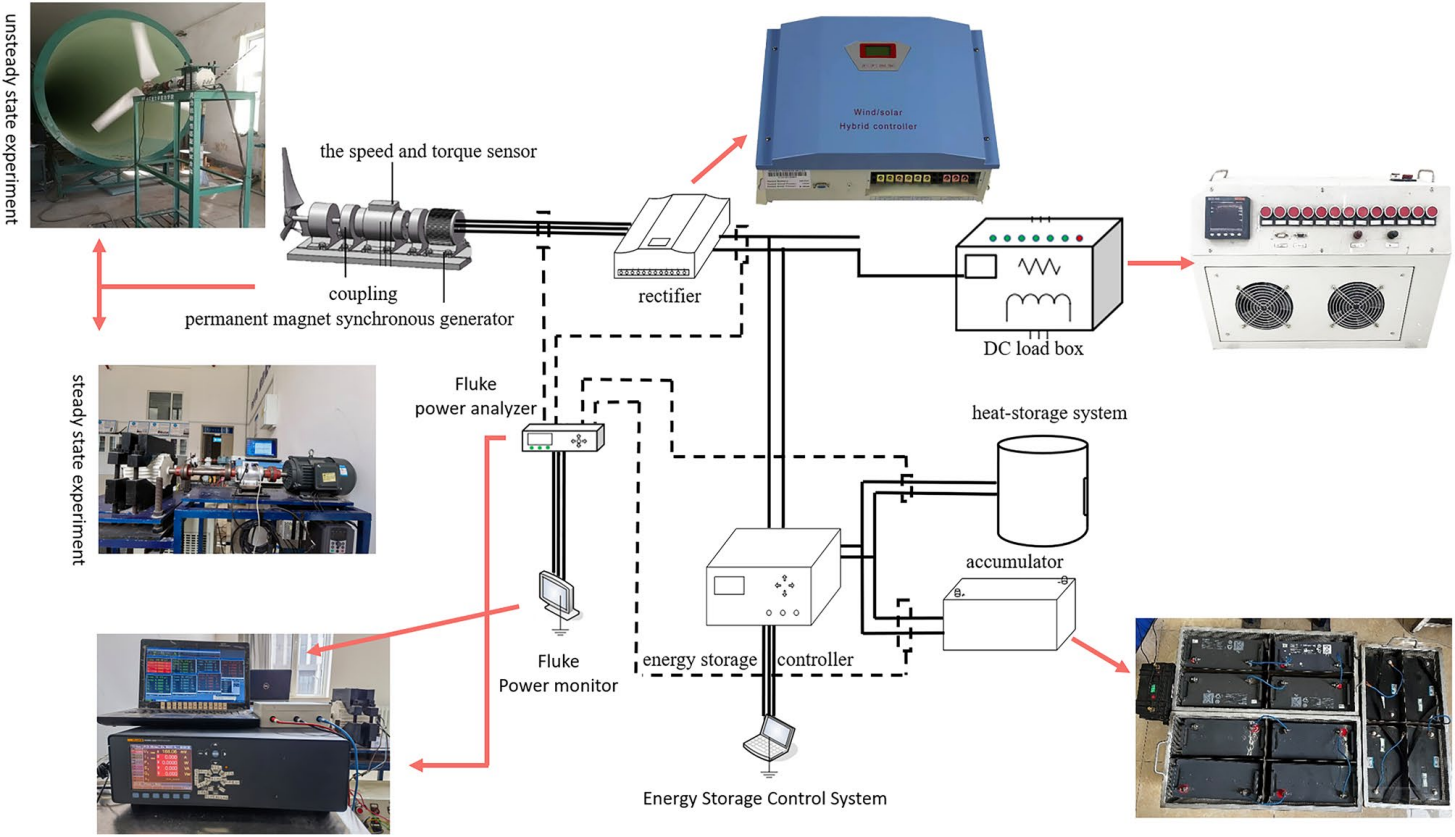
Experimental platform of wind power heat-electric hybrid energy storage.

**Figure 3:**
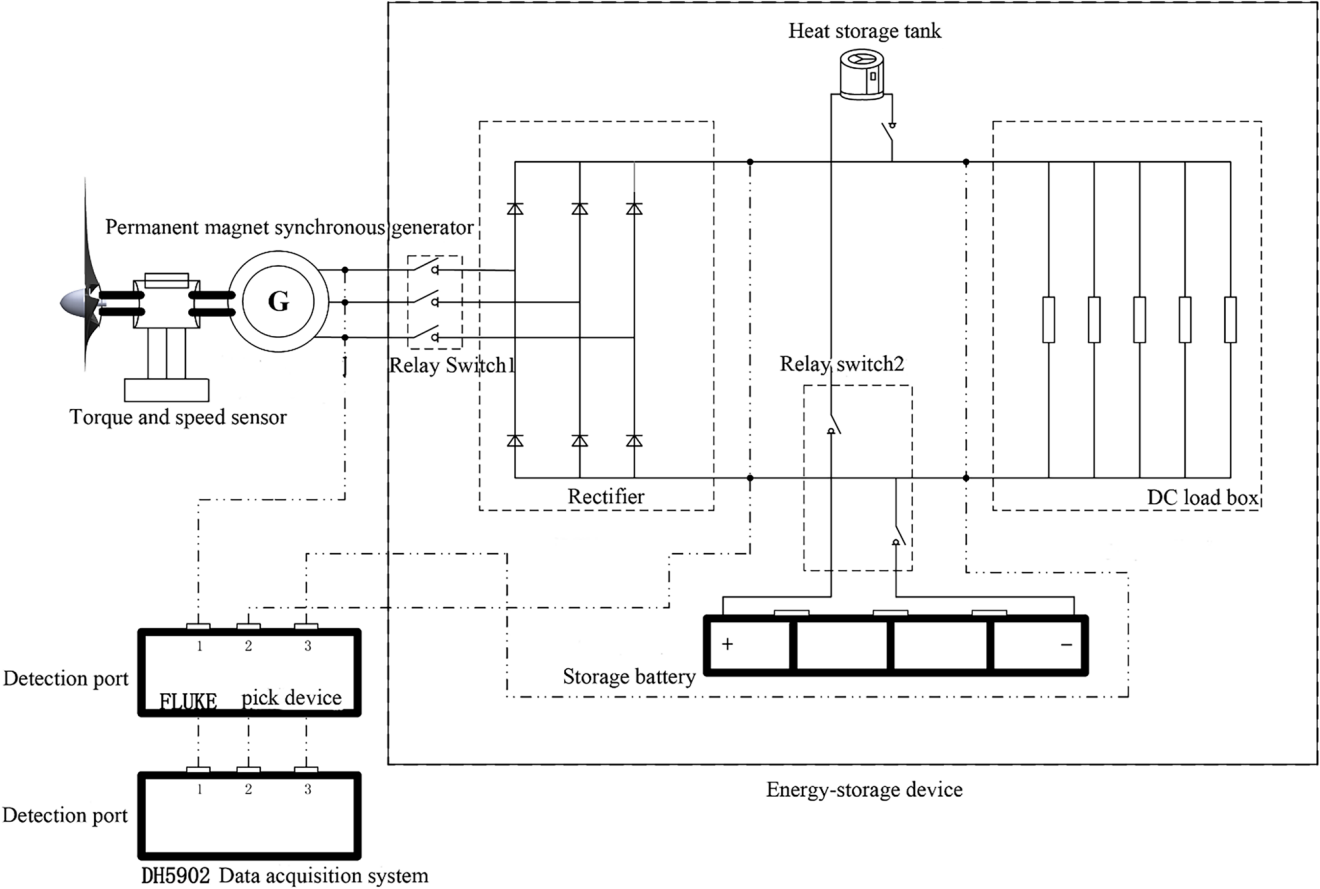
Experimental test circuit diagram.

This experiment relies on the key laboratory of the Ministry of Education of wind energy and solar energy. Based on the above system economic dispatch model, the experimental platform of wind power heat-electric hybrid energy storage is built as shown in Fig. 2. The wind speed is 6–12 m/s, and the speed of the permanent magnet synchronous generator is maintained at 100–600 RPM through the DC load box adjustment. Fluke Norma 5000 power analyzer is used to collect the voltage and current data of the generator, DC load, battery and heat storage system at every 50 RPM. The experimental test circuit is shown in Fig. 3.

The main equipment of the experiment includes wind wheel with rated wind speed of 10m/s and rated output power of 500 W, frequency converter, ZH07-A-100Z torque speed sensor, Fluke Norma 5000 power analyzer, 1KW permanent magnet synchronous generator, rectifier, DC load box, 48V400Ah lithium iron phosphate battery, 48V400Ah lead-acid battery, 48V400Ah lead–carbon battery, 300 W DC heating and heat storage system.

## Analysis of system exergoeconomic characteristics

In order to explore the influence of tip speed ratio, rotational speed and operating conditions on the exergoeconomic characteristics of the system, comparative experiments on the economic characteristics of systems without energy storage, single heat storage, lithium iron phosphate battery energy storage, lead-acid battery energy storage, lead–carbon battery energy storage, lithium-water hybrid energy storage, lead-acid–water hybrid energy storage and lead–carbon-water hybrid energy storage were designed respectively. The frequency converter was adjusted to change the wind speed v, and the operating speed of each system was obtained with v and wind wheel radius R as constraints.

### Exergy efficiency of wind turbine and generator system

**Figure 4:**
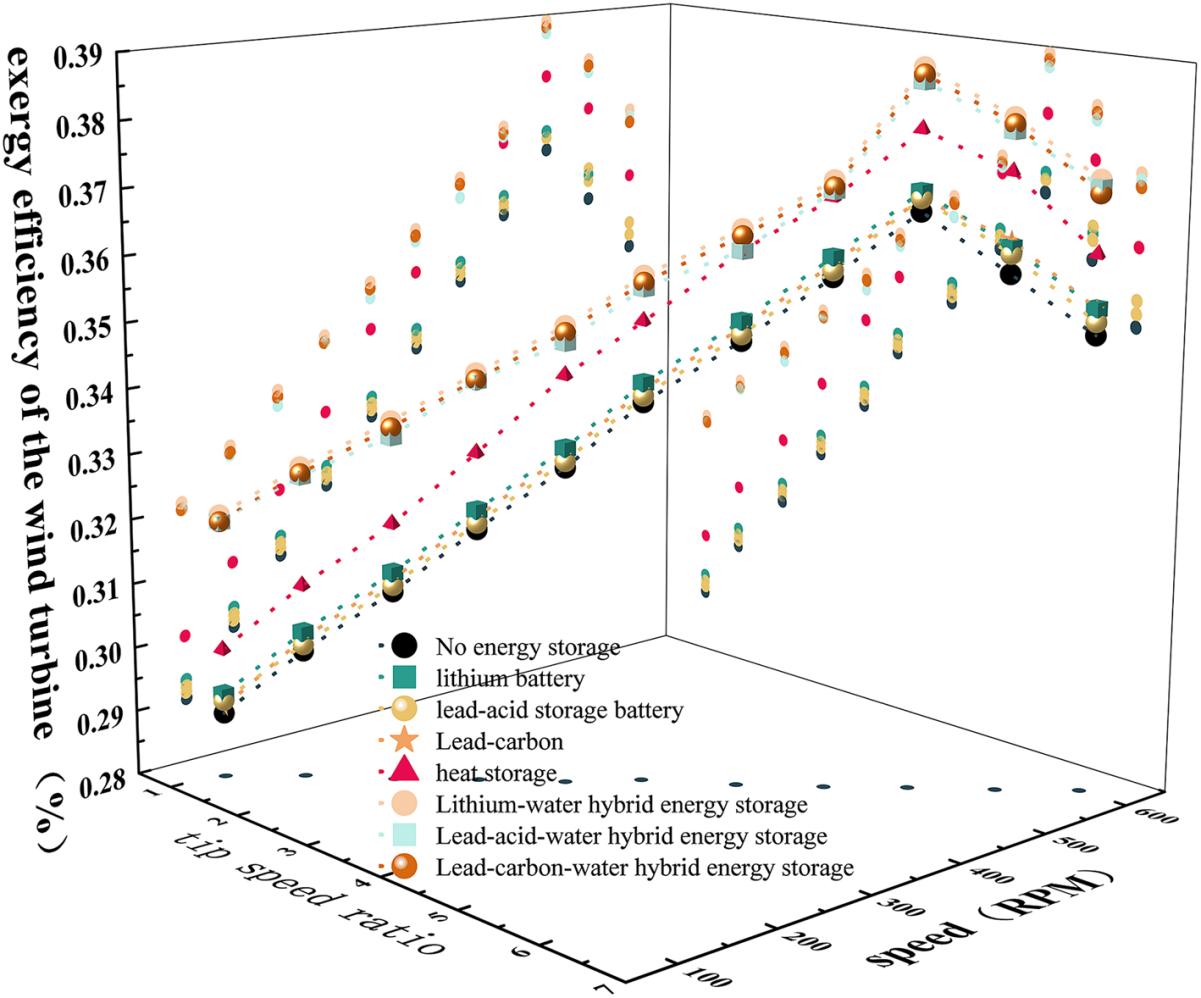
Exergy efficiency of wind turbine system.

**Figure 5:**
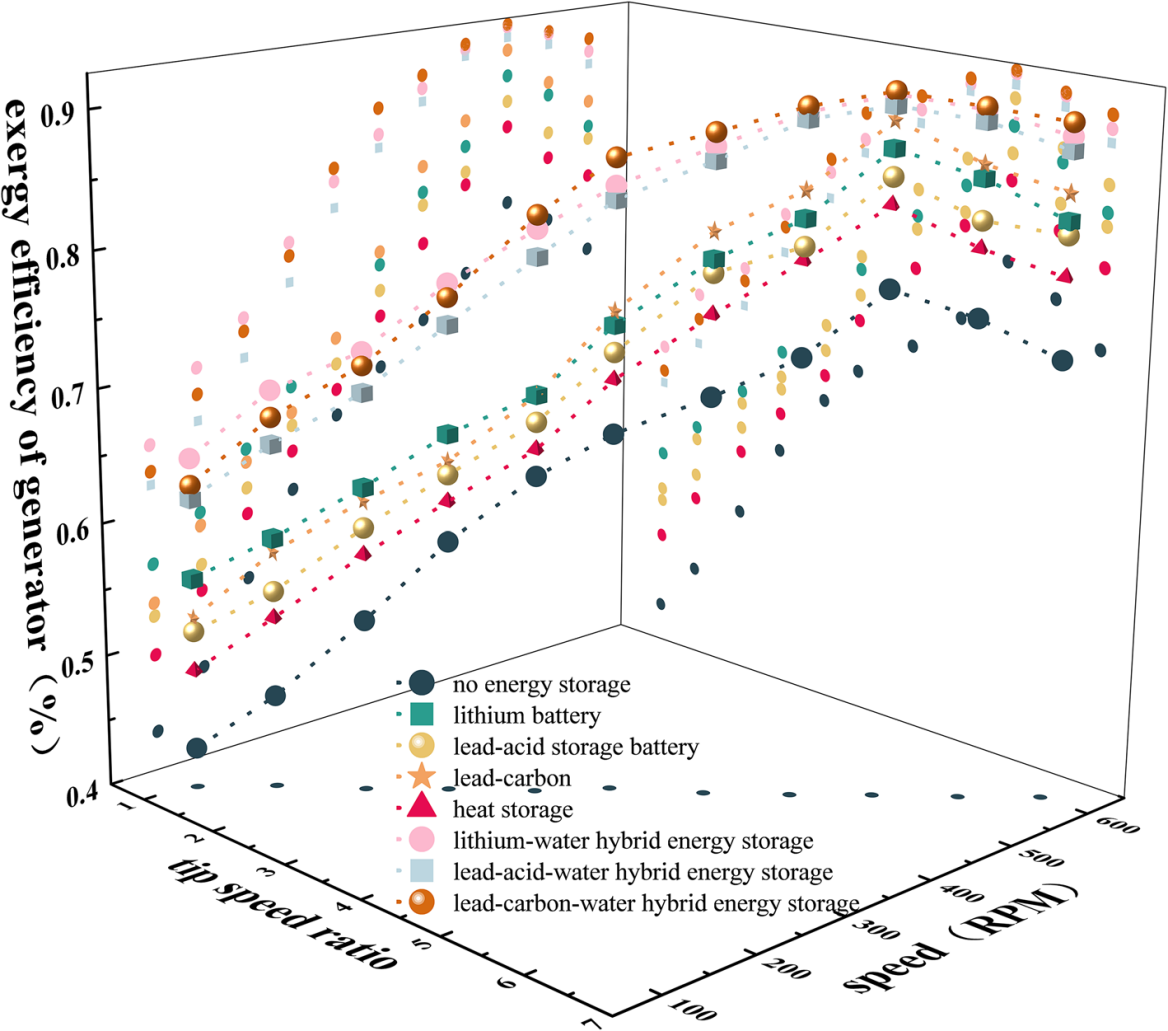
Exergy efficiency of generator system.

Comparing different energy storage systems, the exergy efficiency η1 of the wind turbine system is shown in Fig. 4, and the exergy efficiency η2 of the generator system is shown in Fig. 5. It can be seen that the efficiency η1 of the wind wheel system and the efficiency η2 of the generator system are related to λ, n and different energy storage systems. With the increase of λ and n, η1 and η2 increase first and then decrease.

In the wind-storage hybrid system, the heat storage and battery systems are compared respectively. It can be seen that the heat storage can further improve η1 compared with the battery system, but changing the type of battery has little effect on it. The main reason is that η1 is related to the utilization coefficient of wind energy and has nothing to do with the type of battery, while the addition of heat storage system increases the output power of the wind wheel, thus increasing the value of η1. Comparing the efficiency of the generator system of three kinds of battery energy storage and thermal-electric hybrid energy storage, it can be seen that the η2 of lithium battery is the highest, and the η2 of lithium-water hybrid energy storage is the highest. The main reason is that η2 is related to generator exergy loss, irreversibility of permanent magnet materials and heat dissipation conditions, and the output power quality and speed of the system jointly determine the generator exergy loss.

Therefore, compared with the single energy storage system, the hybrid energy storage system can further improve the power quality of the power generation system, reduce the power loss of the generator, reduce the temperature of the motor, reduce the exergy loss of the motor and increase the efficiency. However, if the motor runs beyond the rated speed, its exergy loss increases, the exergy efficiency decreases, and the battery discharge will cause exergy loss.

### Exergy efficiency of battery system

**Figure 6:**
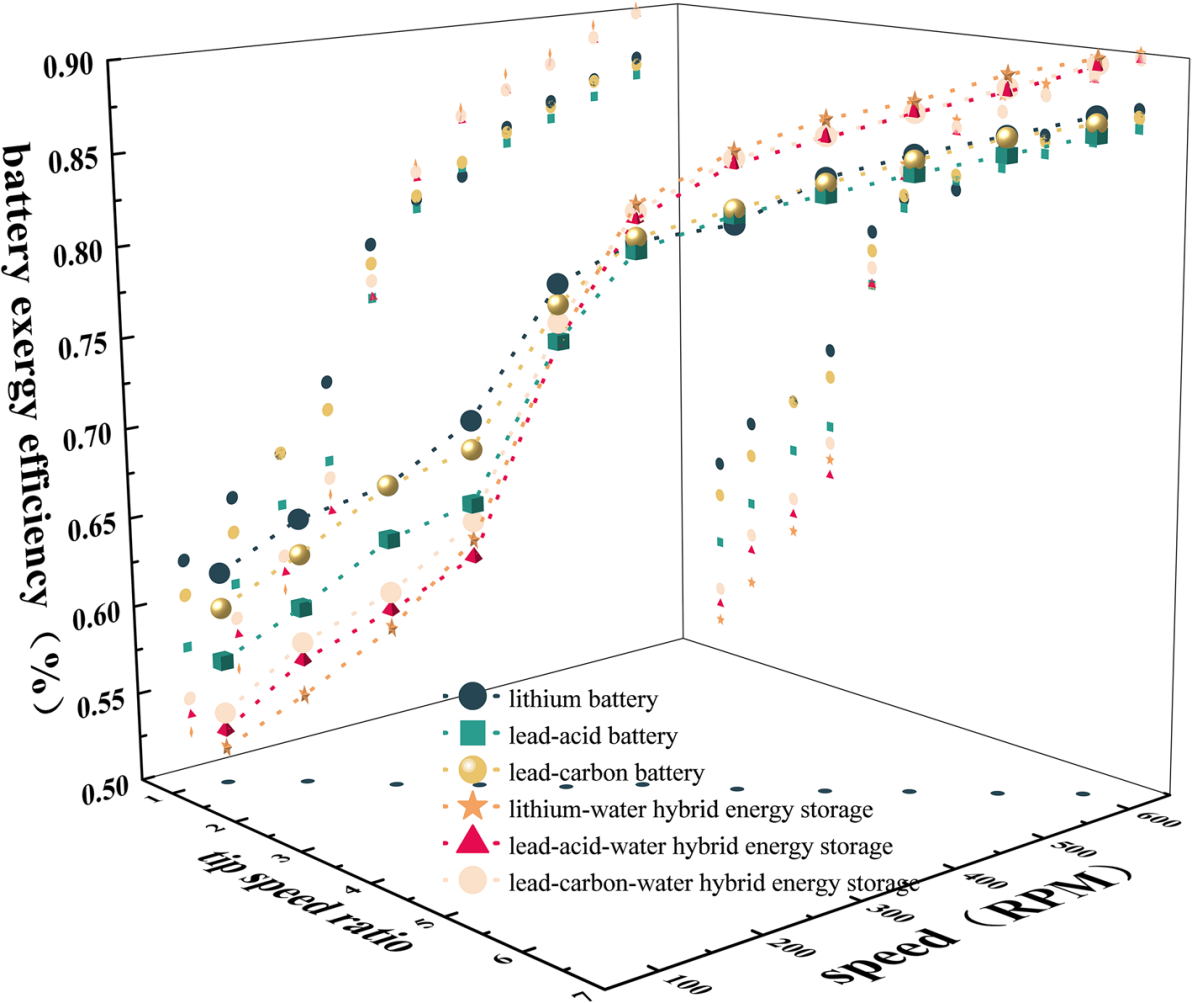
Exergy efficiency of the battery system.

The efficiencies η3 of different types of batteries are shown in Fig. 6. It can be seen that η3 increases linearly with the increase of λ and n. When λ and n are low, the battery is in discharge state, and η3 rises slowly. When λ is 1.08–3.23 and n is 100–300 RPM, the η3 of the battery energy storage system is greater than that of the thermal-electric hybrid energy storage system; when λ is 3.23–6.47 and n is 300–600 RPM, the η3 of the thermal-electric hybrid energy storage system is greater than that of the battery energy storage system. The main reason is that when λ is 1.08–3.23 and n is 100–300 RPM, the battery is in discharge state, and the battery is only powered by load. In the thermal-electric hybrid energy storage system, the battery will simultaneously supply power to the heat storage system and the load, so that the internal heat of the system increases, the exergy loss increases, and the exergy efficiency decreases. When λ is 3.23–6.47 and n is 300–600 RPM, the battery is in the charging state. At this time, the thermal-electric hybrid energy storage system can not only absorb the internal exergy loss of the battery in the charging state, but also improve the exergy efficiency.

Compared with three kinds of battery energy storage and three kinds of thermal-electric hybrid energy storage systems, the η3 of lithium battery is the best, and the η3 of lithium-water hybrid energy storage system is the best. The main reason is that the performance of lithium battery is better than that of lead-acid and lead–carbon batteries. In the same heat dissipation environment, the lithium battery itself has less loss and higher efficiency.

### Exergy efficiency of heat storage system

**Figure 7:**
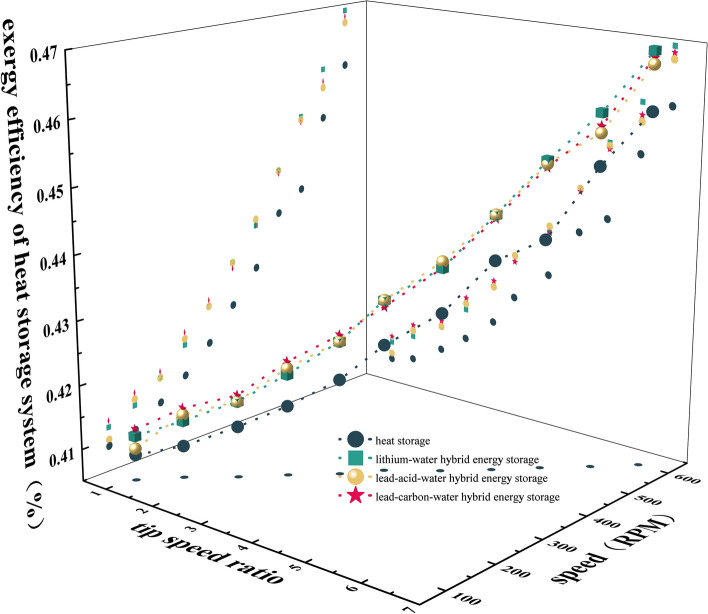
Exergy efficiency of heat storage system.

The exergy efficiency η4 of the thermal-electric hybrid energy storage system is shown in Fig. 7. The system exergy efficiency increases with the increase of λ and n, but the change of battery type has little effect on η4. The main reason is that whether the battery is in discharge or charging state, the heat storage power becomes larger, resulting in faster temperature rise, increased heat absorption of the system, and larger exergy efficiency. However, the type of battery has little effect on the heat storage absorption power and cannot cause temperature change, so the exergy efficiency basically does not change.

### System exergy efficiency

**Figure 8:**
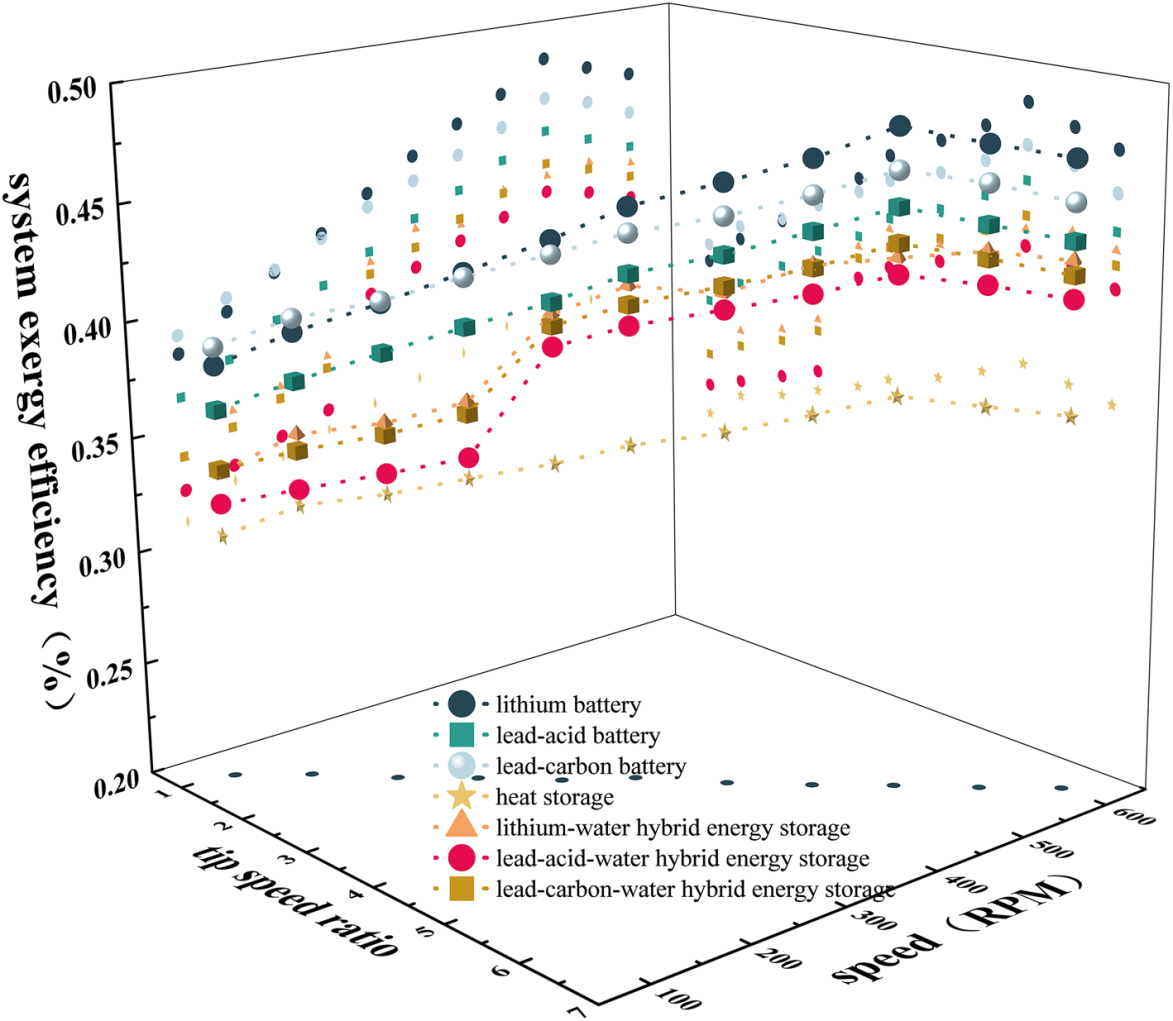
Impact on exergy efficiency.

The system exergy efficiency η is shown in Fig. 8. It can be seen that when λ is 1.07–5.39 and n is 100–500 RPM, η shows an increasing trend. When λ is 5.39–6.46 and n is 500–600 RPM, η shows a decreasing trend. Among them, the maximum value of η of lithium battery energy storage is 47.6%, and the maximum value of η of lithium-water hybrid energy storage is 41.9%. Therefore, the η of battery energy storage system is greater than that of single heat storage and thermal-electric hybrid energy storage system. The thermal-electric hybrid energy storage system with λ of 3.23–6.47, n of 300–600 RPM is more efficient than that with λ of 1.08–3.23 and n of 100–300 RPM. The main reason is that the exergy efficiency η4 of the heat storage system is low. Although the heat storage system can increase η1, η2 and η3 to varying degrees, its own exergy efficiency is low, and η becomes low after adding the heat storage system. When λ is 3.23–6.47 and n is 300–600 RPM, the η3 of the battery charging state is greater than the η3 of the discharge state, which makes the η larger.

### Analysis of system economic characteristics

In order to compare the losses of different energy storage systems more accurately, the optimization direction of maximizing exergy efficiency and minimizing exergetic cost is explored. In this study, the system is evaluated in an exergoeconomic environment.

**Figure 9:**
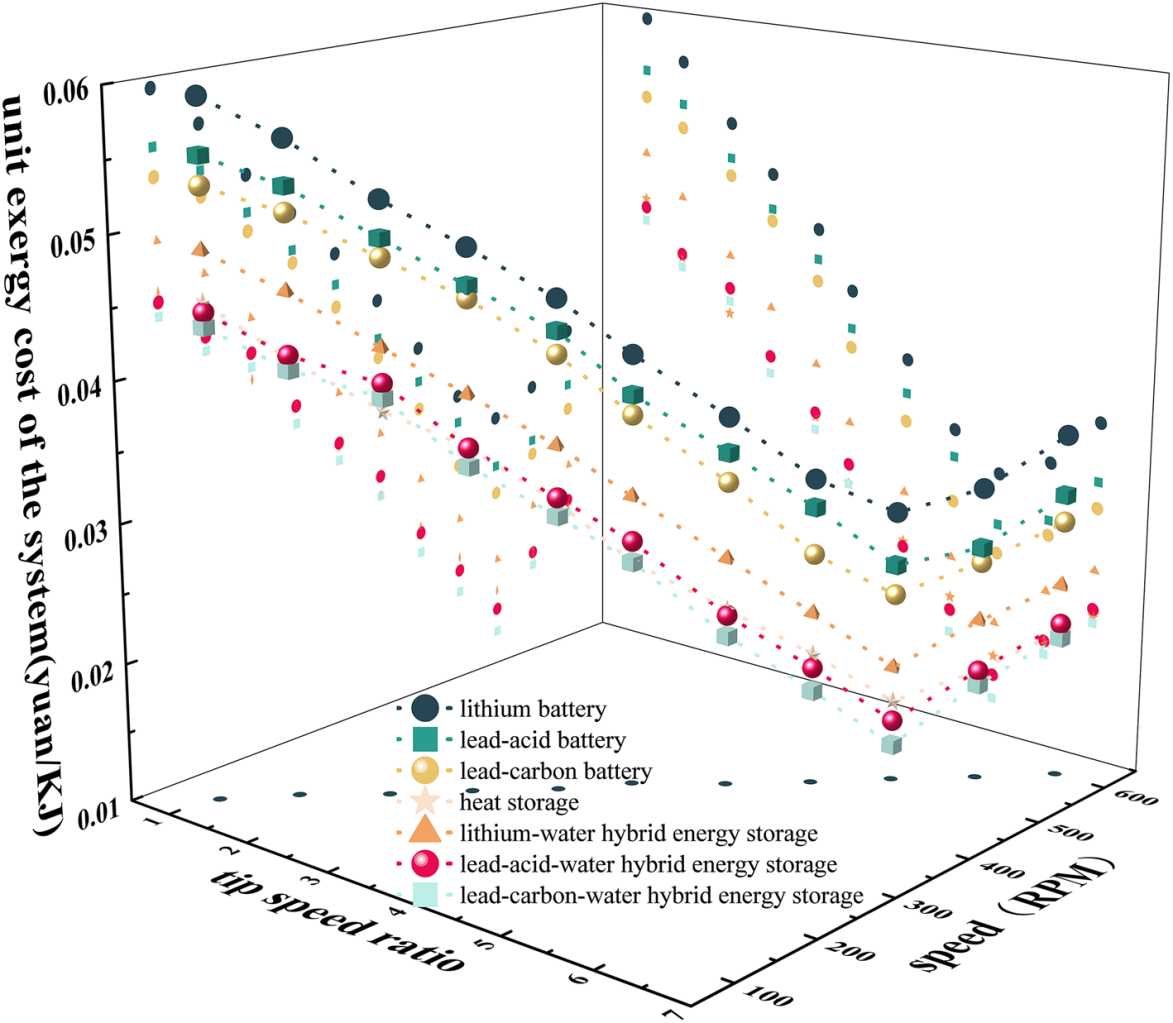
Exergy cost per unit.

The unit cost C_i_ of different energy storage systems is shown in Fig. 9. It can be seen that with the increase of λ and n, C_i_ increases first and then decreases; the C_i_ of the lithium battery energy storage system is higher, while the C_i_ of the lead–carbon-water hybrid system is lower. The main reason is that the lithium battery with lithium iron phosphate as the cathode material is a scarce resource, and its cost is higher than that of lead-acid and lead–carbon batteries, resulting in higher C_i_; in addition, compared with the battery energy storage system, adding heat storage devices can further reduce costs. Therefore, the thermal-electric hybrid energy storage system can effectively reduce the unit cost and improve the energy utilization rate.

## Optimization strategy and multi-objective optimization

### Optimization of system exergy economic characteristics based on RSM

Based on the results of experimental analysis, the response surface methodology (RSM) was used to design the experiment, and the Box-Benhnken central composite design was used to design the experiment.

Three factors, A (wind speed), B (speed) and C (energy storage form), which have significant effects on system exergy efficiency (Y_1_), unit exergy cost (Y_2_) and current harmonic distortion rate (Y_3_), are selected for response surface analysis experiments to maximize system exergy efficiency, minimize unit exergy cost and current harmonic distortion rate as much as possible. The specific test parameter design is shown in Table 1.

**Table 1:** Response surface test design specific parameters.

Design variables	Units	Optimal range
Wind speed (A)	m/s	[6, 12]
Rotate speed (B)	rad/min	[100, 600]
Energy storage form (C)		[1, 3]

The factor C (energy storage form) is explained as follows: the interval [1] is the battery energy storage system, the interval [2] is the heat storage system, and the interval [3] is the thermal-electric hybrid energy storage system.

### Establish response surface model

In this study, the response surface regression analysis was carried out by design expert software, and the following response surface regression model was obtained. The system exergy efficiency, unit exergy cost and current harmonic distortion rate response value are recorded as shown in Table 2.

19 $$\begin{gathered} Y_{1} \left( \% \right) = 31.79 + 0.9625A + 3.61B - 3.15C - \hfill \\ 0.275AB - 0.2000AC + 0.0500BC + 0.5375 \hfill \\ A^{2} - 1.86B^{2} + 9.42C^{2} \hfill \\ \end{gathered}$$

20 $$\begin{gathered} Y_{2} \left( {yuan/KJ} \right) = 0.0264 - 0.0005A - 0.0118B - \hfill \\ 0.0052C - 0.000005AB + 0.0008AC + 0.00003 \hfill \\ BC + 0.0032A^{2} + 0.0067B^{2} + 0.0034C^{2} \hfill \\ \end{gathered}$$

21 $$\begin{gathered} Y_{3} \left( \% \right) = 15.90 + 0.9000A - 3.51B - 0.7575C - \hfill \\ 0.0500AB + 0.0100AC + 0.0450BC + 0.1175 \hfill \\ A^{2} + 2.46B^{2} - 0.5775C^{2} \hfill \\ \end{gathered}$$

**Table 2:** The system exergy efficiency, unit exergy cost and current harmonic distortion rate response value are recorded.

Group	A wind speed (m)	B speed (rad/min)	C energy-storage system	System exergy efficiency (%)	Unit exergy cost (yuan/kJ)	Current harmonic distortion (%)
1	6	100	2	26.5	0.04893	20.96
2	12	100	2	28.9	0.04685	22.88
3	6	600	2	32.6	0.0257	14.17
4	12	600	2	33.9	0.0236	15.89
5	6	350	1	43.6	0.03969	15.11
6	12	350	1	46	0.03796	16.89
7	6	350	3	37.9	0.02631	13.99
8	12	350	3	39.5	0.02785	15.77
9	9	100	1	38.2	0.05326	22.36
10	9	600	1	47	0.02869	15.11
11	9	100	3	31.6	0.04369	20.36
12	9	600	3	40.6	0.02023	13.29
13	9	350	2	31.8	0.02645	15.9
14	9	350	2	31.7	0.0264	15.91
15	9	350	2	31.75	0.02642	15.92
16	9	350	2	31.8	0.02643	15.89
17	9	350	2	31.9	0.02645	15.88

### Response surface analysis

**Figure 10:**
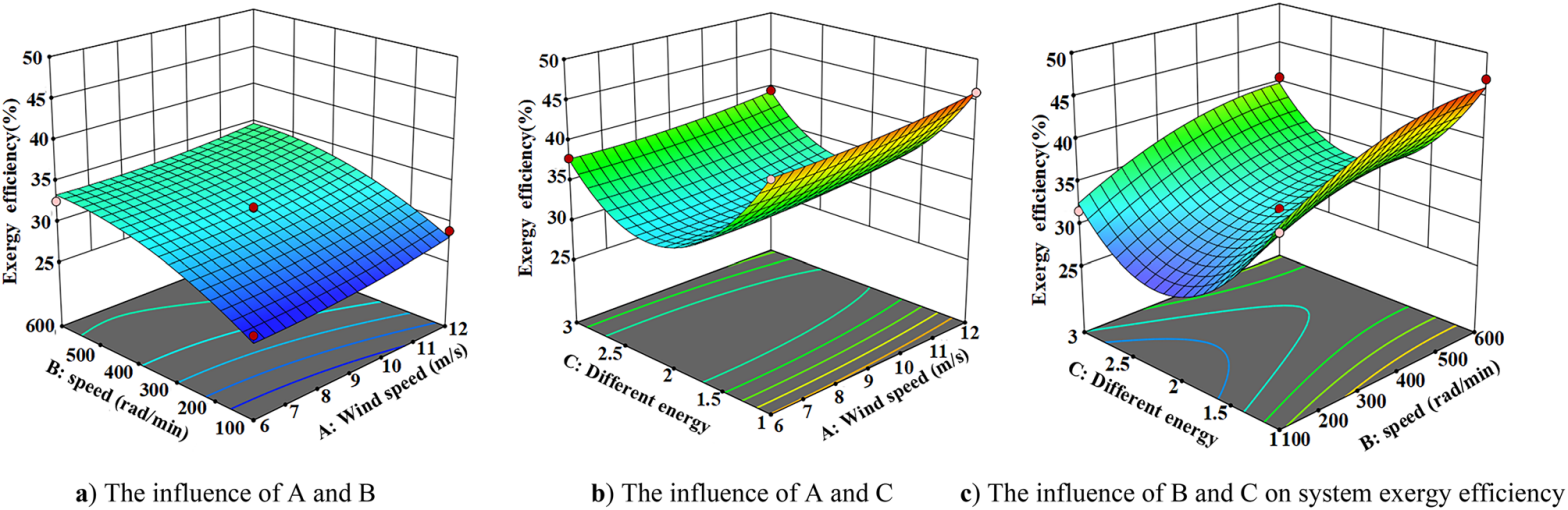
Exergy efficiency 3D response surface map. (**a**) The influence of A and B (**b**) The influence of A and C (**c**) The influence of B and C on system exergy efficiency.

**Figure 11:**
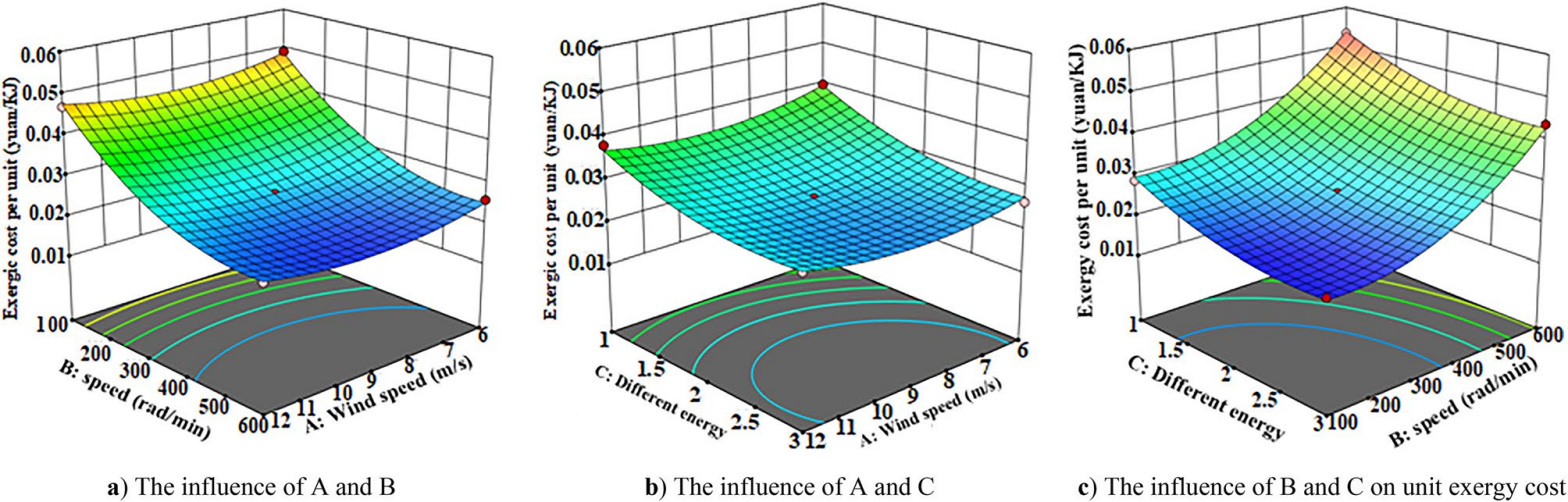
Exergy cost three-dimensional response surface map. (**a**) The influence of A and B, (**b**) The influence of A and C, (**c**) The influence of B and C on unit exergy cost.

**Figure 12:**
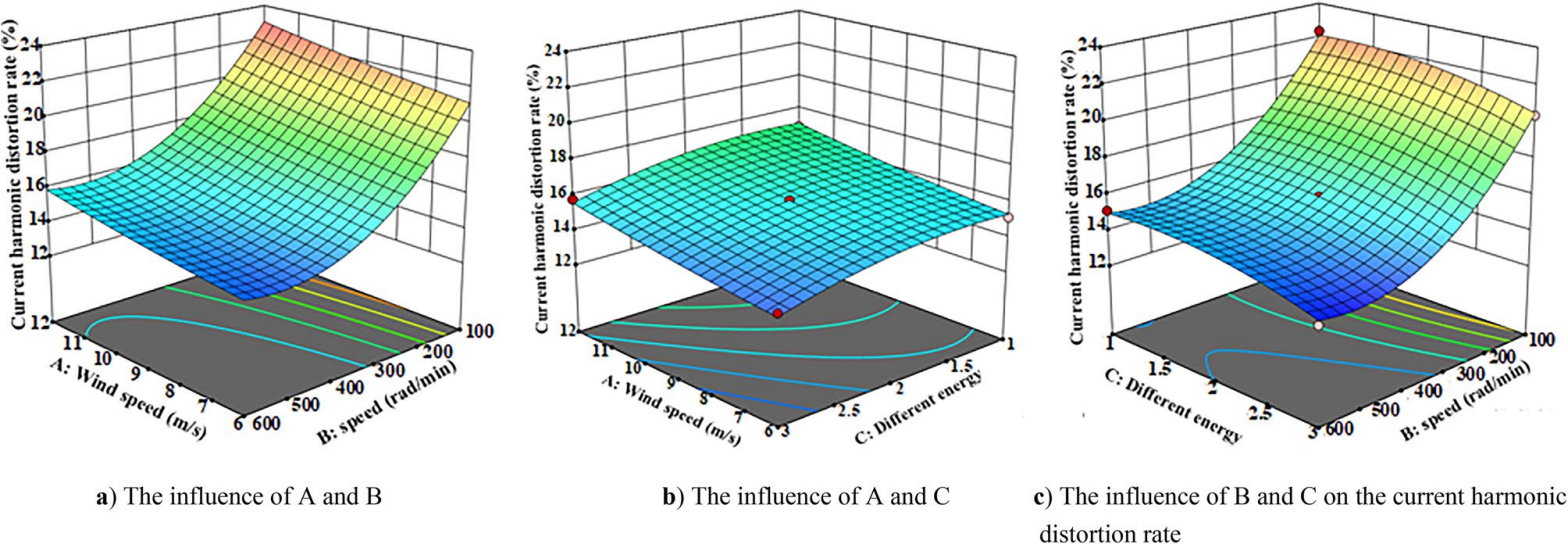
Three-dimensional response surface diagram of current harmonic distortion rate. (**a**) The influence of A and B (**b**) The influence of A and C (**c**) The influence of B and C on the current harmonic distortion rate.

The response surface method is used to analyze the influence of the interaction of different wind speed, speed and energy storage forms on the system exergy efficiency, unit exergy cost and wave distortion rate. The three-dimensional response surface of different influencing factors are obtained according to the regression equation, as shown in Figs. 9, 10, 11 and 12

Figure 10 is the three-dimensional response surface diagram of system exergy efficiency. According to Fig. 10a–c, the speed and different energy storage devices have a significant impact on the system exergy efficiency. With the increase of speed, the system exergy efficiency gradually increases. The efficiency of battery is higher than that of heat storage and thermal-electric hybrid energy storage system. The main reason is that when the load is constant, the power deviation increases with the increase of the speed, and the battery can absorb the excess energy and improve the efficiency of the system. Because the lithium battery has a small polarization internal resistance and less irreversible loss, the efficiency of the lithium battery system is relatively high at the same speed.

Figure 11 is the three-dimensional response surface diagram of unit exergy cost. According to Fig. 11a–c, it can be seen that the speed and different energy storage devices have a relatively large impact on the unit exergy cost. Among them, according to Fig. 11a shows that when the rotational speed is low, the unit exergy cost is higher. Comparing Figs. 10c, 11b, it is found that with the increase of wind speed, the unit exergy cost decreases first and then increases. The reason is that rated wind speed of the wind power system is 10 m/s, below or above the rated wind speed will lead to the wind curtailment of the system, the cost of wind curtailment will increase, and the unit cost will increase.

Figure 12 is the three-dimensional response surface diagram of current harmonic distortion rate. According to Fig. 12a–c, it can be seen that the speed and different energy storage devices have a relatively large influence on the current harmonic distortion rate. It can be seen from Fig. 12a that when the speed is low, the current harmonic distortion rate is high. Comparing Figs. 11c and 12b, it is found that the change of speed has little effect on the current harmonic distortion rate, and the current harmonic distortion rate of the thermal-electric hybrid energy storage system is smaller than that of the battery and heat storage device. The reason is that at low speed, the electromagnetic induction time is short, so that the current cannot completely with the change of magnetic field, and the output current will produce more high-order harmonic components. The thermal-electric hybrid energy storage technology can further absorb and smooth the instantaneous current peak and reduce the current harmonic distortion rate.

Through the analysis of the above wind speed, speed and different energy storage devices on the system exergy efficiency, unit exergy cost and current harmonic distortion rate, the optimal value of the experiment is obtained from the response surface, contour map and regression equation analysis. Under the condition of maximizing the system exergy efficiency and minimizing the unit exergy cost and current harmonic distortion rate, the best working conditions are: wind speed 8.499 m/s, speed 573.562 RPM, system exergy efficiency of thermal-electric hybrid energy storage is 39.776%, unit exergy cost is 0.02 yuan/KJ, current harmonic distortion rate is 13.29%.

### Optimization strategy

In this paper, it is assumed that the loss of the generator and the loss of the battery charge and discharge process are constant, and the simulation model of the thermal-electric hybrid energy storage wind power system is established.

In the simulation system, the wind speed of 6 to 12 m/s matched with the experiment is input, and the three-phase current emitted by the 1 KW permanent magnet synchronous generator is rectified and connected to the 48 V/100Ah lithium battery, load and heat storage device respectively. In the case of not affecting the simulation effect, the simulation time is set to 1 s.

**Figure 13:**
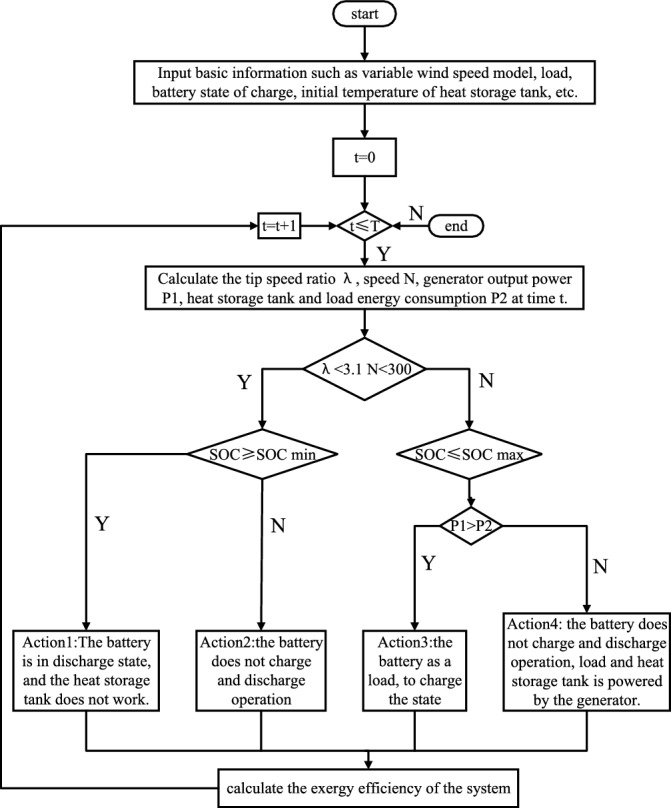
Optimal scheduling strategy.

By simulating the wind storage hybrid system with different wind speed, speed and tip speed ratio, based on the the system exergy efficiency and the state of charge of the battery, the charge and discharge status of different energy storage devices and batteries is changed to improve the power quality of the wind power system. The thermal-electric hybrid energy storage wind power system exergy efficiency reduces the unit exergy cost, and the optimization strategy process is shown in Fig. 13:

Action 1, 2: When λ < 3.1n < 300RPM, and the heat storage system is not involved in the work. When the storage capacity of the battery is greater than the minimum capacity of the battery, the battery is in the discharge state, and the battery and the generator supply power to the load at the same time. If the battery has no storage capacity, there is no charge and discharge operation.

Action 3, 4: When λ > 3.1n > 300RPM, and the heat storage system participates in the work. The power generated by the generator is supplied to the load and the heat storage system, and the battery capacity is less than its maximum capacity, and the battery is in the charging state. If the battery is full capacity, there is no charge and discharge operation.

### Multi-objective optimization

In the multi-objective optimization algorithm^48^, this paper selects the common multi-objective algorithm multi-objective particle swarm optimization (MOPSO), multi-objective grey wolf (MOGWO), multi-objective white shark (MOWSO) for comparison.

### Determine the objective function and constraint conditions

In this paper, the objective functions of the real factors of system exergy efficiency Y_1_, unit exergy cost Y_2_, current harmonic distortion rate Y_3_, wind speed A, speed B and different energy storage forms C are obtained by RSM as follows:

22 $$\begin{gathered} {\text{Y}}_{{1}} {\text{(\% ) = 66}}{.8418 - 0}{\text{.5025A + 0}}{\text{.038154B - 40}}{\text{.29C - 0}}{\text{.000367A*B}} \hfill \\ { - 0}{\text{.066667A*C + 0}}{\text{.0002B*C + 0}}{\text{.060278A}}^{{2}} { - 0}{\text{.00003B}}^{{2}} { + 9}{\text{.4175C}}^{{2}} \hfill \\ \end{gathered}$$

23

24 $$\begin{gathered} {\text{Y}}_{{3}} {\text{(\% ) = 23}}{.1127 + 0}{\text{.088333A - 0}}{\text{.041334B + 1}}{\text{.4895C - 0}}{\text{.000067A*B}} \hfill \\ { - 2}{\text{.26557e}}^{{ - 16}} {\text{A*C + 0}}{\text{.00018B*C + 0}}{\text{.013056A}}^{{2}} { + 0}{\text{.000039B}}^{{2}} { - 0}{\text{.5775C}}^{{2}} \, \hfill \\ \end{gathered}$$

The optimization goal is to obtain a set of optimal parameter combinations of system exergy efficiency Y_1_, unit exergy cost Y_2_ and current harmonic distortion rate Y_3_, so that the system exergy efficiency Y_1_ is maximized, and the unit exergy cost Y_2_ and current harmonic distortion rate Y_3_ are minimized. According to the above analysis, the objective function of the wind-storage hybrid system is:

25 $$\left\{ \begin{gathered} (A,B,C) = \arg \min \left[ { - Y_{1} ,Y_{2} ,\left. {Y_{3} } \right]} \right. \hfill \\ s.t.A \in (6,12),B \in (100,600),C \in (1,3) \hfill \\ \end{gathered} \right.$$

In the formula,$(A,B,C)$ represents the optimal combination of wind speed, speed and different energy storage forms when the system exergy efficiency, unit exergy cost and current harmonic distortion rate reach the minimum. $A \in (6,12),B \in (100,600),C \in (1,3)$ represents the constraint conditions of the optimization problem, which represent the range of wind speed, speed and different energy storage forms respectively.

### Optimization results and evaluation

**Figure 14:**
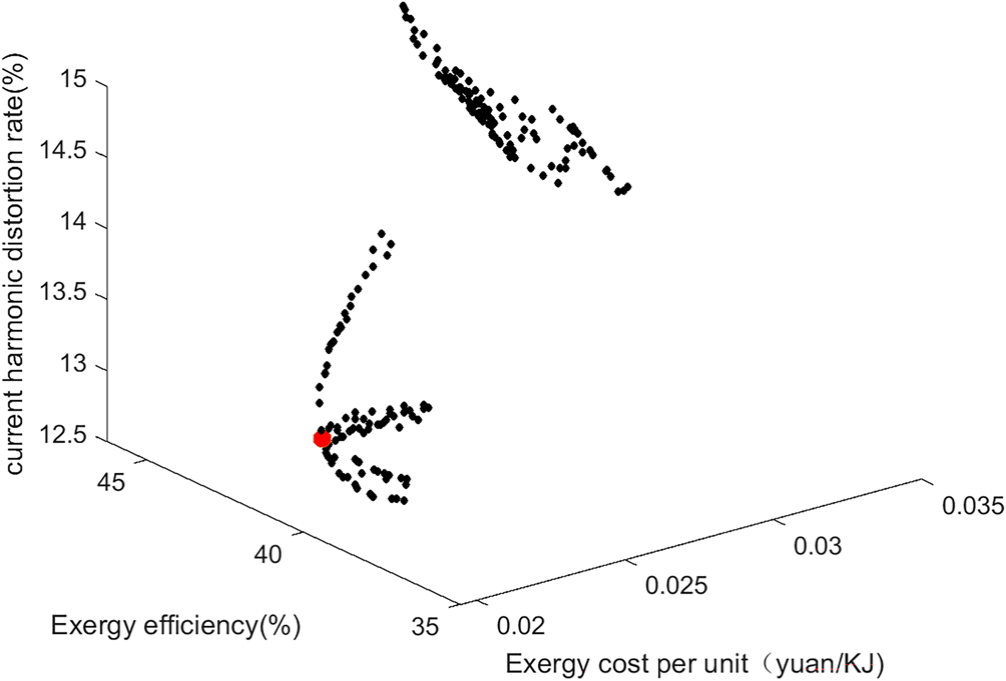
MOGWO.

**Figure 15:**
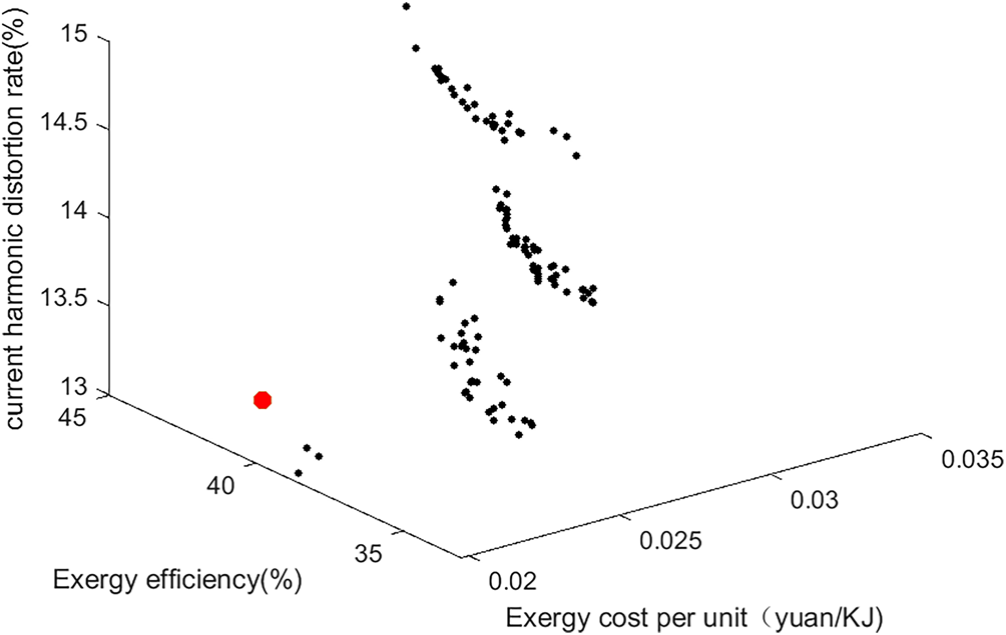
MOGWO.

**Figure 16:**
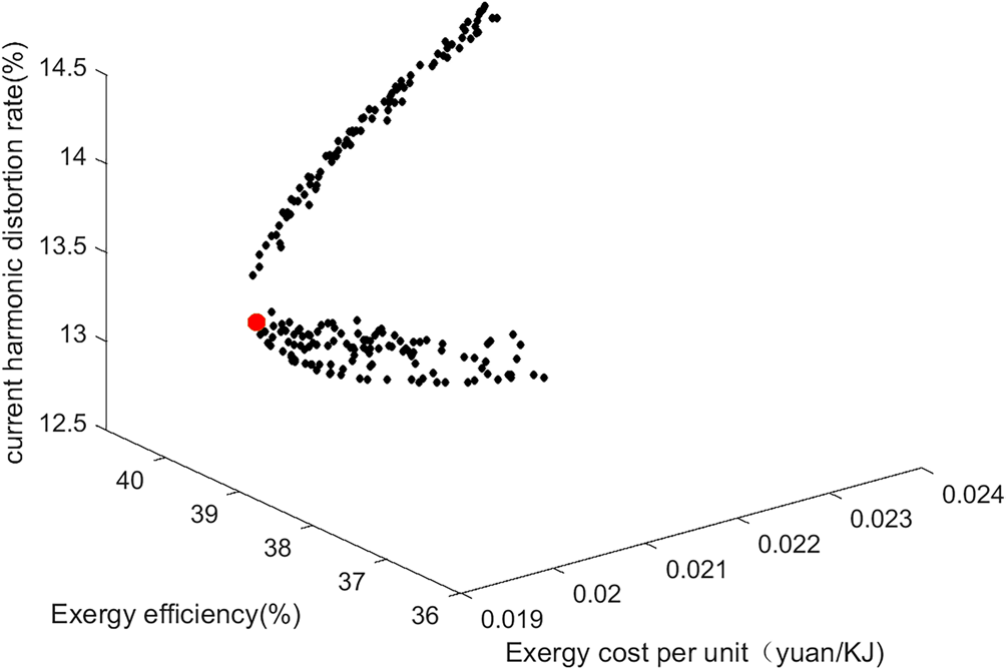
MOWSO.

In this paper, the relevant parameters of the thermal-electric hybrid energy storage system are loaded into the three multi-objective algorithms of MOPSO, MOGWO and MOWSO, and the relevant parameters of the algorithm are set. Due to the large difference in the magnitude of the constraint system efficiency Y_1_, the unit cost Y_2_ and the current harmonic distortion rate Y_3_, the weight (0.3, 0.6, 0.1) is normalized. According to the dominant relationship of the algorithm characteristics, the historical optimum (pbest) and the global optimum (gbest) of each particle are determined. After 200 iterations, the results are shown in the Figs. 14, 15 and 16. Figure 14 shows MOPSO. Figure 15 shows MOGWO. Figure 16 shows MOWSO. Each point in the figure represents three characteristics including wind speed, speed and different energy storage forms. The corresponding coordinates represent the system exergy efficiency, unit exergy cost and current harmonic distortion rate under this condition. The red particles in the figure are the optimal solution, as shown in the Table 3.

**Table 3:** Three multi-objective optimal solutions.

	Wind speed, m/s	Rotate speed, rpm	Energy storage form	Exergy efficiency %	Exergic cost per unit, yuan/KJ	Current harmonic distortion rate, %
MOPSO	8.69	548.76	3	39.65	0.02	13.18
MOGWO	9.3	557.8	3	39.78	0.02	13.36
MOWSO	8.84	549.84	3	39.68	0.02	13.21
Experiment	8.49	573.56	3	39.77	0.02	13.29

It can be seen that under the above constraints, the energy storage form is a thermal-electric hybrid energy storage system, the exergy efficiency is increased by 10%, the unit exergy cost is reduced by 0.03 yuan/KJ, and the current harmonic distortion rate is reduced by 8%. The optimal solution is not much different from the optimal solution found in the experiment. The results of the above algorithm based on standard deviation evaluation are shown in the Table 4. It can be seen that the minimum standard deviation of MOGWO is close to the optimal value, and the effect is the best.

**Table 4:** Standard deviation evaluation results.

	Exergy efficiency	Exergic cost per unit	Current harmonic distortion rate
MOPSO	41.63	0.03	13.99
MOGWO	36.93	0.02	14.28
MOWSO	39.55	0.02	13.48

### Strategy and algorithm verification analysis

**Figure 17:**
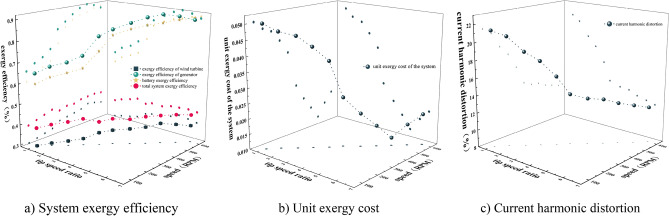
The scheduling strategy and optimization algorithm. (**a**) System exergy efficiency, (**b**) Unit exergy cost, (**c**) Current harmonic distortion.

In order to verify the effectiveness of the above scheduling strategy and algorithms, the influence of changing the charging and discharging state of energy storage device and battery with different tip speed ratio on each subsystem, system exergy efficiency, unit exergy cost and current harmonic distortion rate in the simulation system is shown in Fig. 17.

According to the scheduling strategy and optimization algorithm Fig. 17a–c, it can be seen that the increase trend of exergy efficiency η1, exergy efficiency η2 and exergy efficiency η3 of the wind turbine system with λ of 1.08–3.23 is smaller than that of λ is 3.23–6.46. However, the system exergy efficiency η is smaller when λ is 3.23–6.46 than when λ is 1.08–3.23 and when λ is 3.23–6.46, the unit exergy cost and current harmonic distortion rate decrease significantly.

The main reason is that when λ is 1.08–3.23, the heat storage system does not participate in the work and the battery is in a discharge state, resulting in smaller η1, η2, η3 and larger η; when λ is greater than 3.23, the heat storage system participates in the work. At this time, the battery is in charged state, and the generator is powered by load, heat storage and battery. Due to the low exergy efficiency of the heat storage device, the state η value is small.

In this paper, the scheduling strategy and optimization algorithm are used to obtain the system efficiency, unit exergy cost and current harmonic distortion rate under different working conditions. The scheduling strategy first avoids the situation that η2, η3 and η are low when t λ is small. Second, when λ is large, the generator is powered by load, heat storage and battery. At this time, the heat storage device is added to reduce the unit exergy cost and improve the economic operation of the system. The internal exergy loss of the battery can be absorbed, the current harmonic distortion rate can be reduced, the power quality can be improved, the battery exergy efficiency can be improved, and the system exergy efficiency can be improved.

## Conclusions

To realize the optimal configuration of the thermal-electric hybrid energy storage wind power system, it is necessary to consider not only the social economy but also the natural environment, and consider the energy efficiency of the wind turbine and reduce the cost from the perspective of the whole life cycle.

In this study, the wind-electric-heat hybrid energy storage system is studied by combining experiment and simulation, and the economic mathematical model of wind power hybrid energy storage system is established to improve the power quality and efficiency of the system and reduce the unit exergy cost. The distribution of exergoeconomic characteristics of different energy storage systems is as follows:

1. The efficiency η1 of the wind wheel is related to the wind energy utilization coefficient and has nothing to do with the battery type. The addition of the heat storage system increases the output power of the wind wheel and increases the value of η1. The performance of lithium battery is better than that of lead-acid and lead–carbon batteries. In the same heat dissipation environment, the lithium battery itself has less loss and higher η3 efficiency.
2. When λ is 1.08–3.23 and n is 100–300 RPM, the battery exergy efficiency η3 is larger; when λ is 3.23–6.47 and n is 300–600 RPM, the η3 of the thermal-electric hybrid energy storage system is larger; the unit cost of thermal-electric hybrid energy storage system is less than that of single battery system. Therefore, when λ and n are large, adding a heat storage system can absorb the internal exergy loss of the battery charging state, and also improve the system exergy efficiency and reduce the unit exergy cost.
3. RSM is used to analyze the power quality and exergoeconomic characteristics of the system, and the exergoeconomic characteristics of the system are optimized with the multi-objective evaluation indexes of system efficiency, unit exergy cost and current harmonic distortion rate. The results show that the exergy efficiency of the thermal-electric hybrid energy storage system is increased by 10%, the unit exergy cost is reduced by 0.03 yuan/KJ, and the current harmonic distortion rate is reduced by 8%. The optimal working conditions under the system are wind speed 8.499 m/s, speed 573.562 RPM, system exergy efficiency 39.776%, unit exergy cost 0.02 yuan/KJ, and current harmonic distortion rate 13.29%.
4. An optimization strategy based on the system exergy efficiency and the state of charge of the battery is proposed to change the energy storage device of the system and the charging and discharging state of the battery. The scheduling strategy can avoid the situation of low η2, η3 and η when λ is small. When λ is large, the unit exergy cost and current harmonic distortion rate can be further reduced, the power quality of the wind power system can be improved, and the exergy efficiency of the thermal-electric hybrid energy storage wind power system can be improved.
5. The author will use the exergy analysis method which combines the non-equilibrium thermodynamics and finite-time thermodynamics of network characteristics to study the wind power hybrid energy storage from the perspective of flow, and study the whole life cycle of wind power hybrid energy storage based on exergy economics.

## Data Availability

This paper is based on the research results of the Key Laboratory of Wind and Solar Energy Technology, Ministry of Education, College of Energy and Power, Inner Mongolia University of Technology. However, the datasets generated and analyzed by the experiment during the current study period cannot be publicly available. The datasets that support the findings of this study are available from the corresponding author upon reasonable request.
